# Recovery of the breast screening programme following pandemic-related delays: Should we focus on round length or uptake?

**DOI:** 10.1177/09691413211066476

**Published:** 2022-06

**Authors:** Stephen W. Duffy, Sue Hudson, Daniel Vulkan, Thomas E. Duffy, Kathie Binysh

**Affiliations:** 1 105714 Wolfson Institute of Population Health, Queen Mary University of London, London, UK; 2 Peel & Schriek Consulting Limited, London, UK; 3 Research Department of Behavioural Science and Health, 4919 University College London, London, UK; 4 Head of Screening, 53249 NHS England & Improvement (London), London, UK

**Keywords:** breast screening, round length, uptake

## Abstract

**Objectives:**

The NHS Breast Screening programme is recovering from the hiatus in screening in 2020 due to the COVID-19 pandemic. Currently, open rather than timed invitations are issued, which leads to lower uptake but more rapid coverage of the eligible population by invitation and therefore closer adherence to a round length of 3 years. We aimed to estimate the likely effect on numbers of cancers detected at incident screens of a range of round lengths and uptake rates.

**Methods:**

We assumed exponential distributions of time to incidence of preclinical screen-detectable cancer and of time to progression thereafter to symptomatic clinical disease. We derived numerical values of these, along with screening sensitivity, from published research results and statistics from the NHS Breast Screening programme. These were used to calculate numbers of cancers detected at incident screens at ages 51–70 by round length and uptake rates.

**Results:**

We found that in a homogeneous population of cancers, a 4-year round length with uptake of 62%, as observed with timed appointments in London before the pandemic, would result in 295 cancers screen detected per 10,000 invited, compared to 222 cancers with a 3-year round and uptake of 46%, as observed in London during the recovery period. Similar results were found when we posited two populations, one of rapidly progressing and one of slowly progressing cancers.

**Conclusions:**

It may be more productive in terms of early detection to focus on uptake rather than round length in the programme's recovery from the pandemic.

## Introduction

The national NHS breast screening programme in England (NHSBSP) invites around 3,000,000 asymptomatic women for screening each year and detects around 19,000 breast cancers per year, accounting for the detection of around 40% of the total breast cancers diagnosed annually.^1,2^ Cancers detected through the programme are on average at an earlier stage at the time of detection and have a better outcome in terms of mortality and morbidity.^3^ An Independent Review, jointly commissioned by Cancer Research UK and the Department of Health (England) in October 2012 concluded that “The breast screening programmes in the United Kingdom, inviting women aged 50–70 every 3 years, probably prevent about 1300 breast cancer deaths a year”.^4^ In Sir Mike Richards’ review of adult screening services, this number was updated to 1700 deaths prevented per year, due to the increased annual screening activity since the independent review.^5^

As a result of the Covid 19 pandemic most routine breast screening was paused for some weeks, resulting in women's invitations for routine screening being delayed, although throughout lockdown screening continued for women at the highest risk. When routine screening restarted there was a significant backlog of invitations. In addition, infection control requirements meant that screening services were only able to operate at a reduced capacity. There is also the possibility of a reluctance to attend for screening for fear of exposure to infection.

To achieve the maximum population benefit from breast screening requires recovery of the national standards that have been impacted by the pandemic, notably screening coverage, as rapidly as possible. Coverage is a function of both timely invitation (round length) and invitation uptake. There are several possible strategies for achieving programme recovery but central to deciding the optimum strategy is the question of how these two important parameters should be prioritised, in order to preserve the maximum population benefit during this period and beyond.

A timed appointment has been shown to be an effective way of achieving high uptake of screening, with timed (fixed) screening appointments increasing participation by around 20% (in absolute terms) in comparison to open invitations.^6^ However, fixed appointments can lead to many wasted appointments because only around 60%-70% of women invited attend. To maximise utilising of slots, women can be offered an ‘open’ invitation whereby they are able to select their own timing. This results in a lower overall uptake of invitation but fewer wasted appointment slots. Because the slots can be utilised more efficiently (and because of the lower uptake) more women can be invited in the same time period using open invitations; this enables a service to recover the Covid-19 backlog more quickly, hence restoring a 3-year round length more rapidly in terms of invitation, at the cost of lower uptake.

To explore this question we considered the impact on cancer detection at incident screens for differing round lengths and levels of uptake.

## Methods

The harvest of cancers at an incident screen for breast cancer depends on:

- the number of persons attending for screening;
- the sensitivity of the screening test;
- the rate of transition from preclinical to symptomatic disease (the inverse of the mean sojourn time, MST);
- the incidence of preclinical breast cancer; and
- the time since the last screen (screening interval).

### Homogeneous population with respect to progression

If we denote sensitivity by S, the incidence of preclinical disease by λ_1_, transition from preclinical to symptomatic disease by λ_2_ and the screening interval by t, the expected rate of cancers at an incident screen with participation probability p can be estimated as:

$$R=\frac{\;pS\lambda_{1}(e^{-\lambda_{1}t}-e^{-\lambda_{2}t})}{\left(\lambda_{2}-\lambda_{1})(1-(1-S)e^{-\lambda_{2}t}\right)}$$

This formula follows the approach of Day and Walter,^7^ except that we assumed an exponential distribution of time to incidence of preclinical disease, rather than a uniform annual incidence. A formal proof of the formula is given in the online Appendix. From the literature, λ_2_ can be estimated as approximately 0.25 for ages 50–69, corresponding to a 4-year MST^8^ As regards sensitivity, one approximation sometimes used is to assume that the interval cancers in the first year after a screen are the cancers missed by that screen. If we use published screen detected and interval cancers from the NHS Breast Screening Programme, this gives as an estimate of sensitivity, S = 0.92.^9^ We then constrained λ_1_ as 0.0040 (i.e. four per thousand per year), in order to give approximately 8 cancers detected per thousand screened with a three-year interval, as observed at incident screens in the NHS Breast Screening programme,^1^ when combined with the above values for S and λ_2_.

We then calculated R for intervals of 3 to 7 years, and uptake rates of 46%, 50%, 60%, 62% and 70%. There was specific interest in 46% and 62% uptake, as 46% has been observed in the current recovery period in London for open invitations and 62% immediately prior to the pandemic for timed invitations. This was then multiplied by the number of incident screens expected from ages 51 to 70, following a prevalent screen at age 50, for each interval: six screens for a 3-year interval (ages 53, 56, 59, 62, 65, 68), five screens for a 4-year interval (54, 58, 62, 66, 70), four screens for a 5-year interval (55, 60, 65, 70), and so on, to give the total number of incident screen cancers detected in the current screening age range, for each interval and uptake rate. We considered only the incident screens as the harvest of cancers at the universal prevalent screen at age 50 would be the same regardless of round length.

We also approximated the total number of cancers occurring from age 50 to age 71 per woman invited (again excluding prevalent screen cancers at age 50 since these would be the same), as

$$T=\overset{21}{\underset{0}{\int }}\;\lambda_{1}e^{-\lambda_{1}t}=1-e^{-21\lambda_{1}}$$

The expected numbers of interval cancers arising were calculated by subtraction of the total screen-detected cancers from the total number of cancers occurring among participants.

### Mixture of fast-growing and slow-growing tumour populations

We then postulated two populations, one of slow-growing cancers with MST 5 years, and therefore a rate of transition from preclinical to clinical disease of λ_2_ = 0.2, and the other of faster growing cancers with MST 2.5 years, and therefore a rate of transition λ_2_ = 0.4. In order to give approximately 8 cancers per thousand screened at incident screens at an interval of three years, the underlying incidence rates (λ_1_) of the two types of cancers were constrained to be 0.00284 and 0.00126, respectively. We then repeated the calculations for intervals of 3–7 years, and uptake rates of 46%, 50%, 60%, 62% and 70%, as before.

## Results

### Homogeneous population with respect to progression

The estimated numbers of cancers incident screen-detected in a population screened over the age range 51–70 are shown in Table 1. Numbers are given per 10,000 women invited. Results indicated for example that a greater benefit in terms of screen detection would accrue from screening every four years with 62% uptake (295 cancers screen-detected between the ages of 51 and 70), than screening every three years with 46% uptake (222 cancers screen-detected). The benefit of increased uptake was estimated to be outweighed by the longer round length of between 6 and 7 years. Correspondingly, interval cancer incidence generally increased with increasing round length, and numbers of cancers in non-attenders were not dependent on round length, but decreased with increasing participation rates. Consequently, total symptomatic cancer numbers were lower, for example, for 62% uptake at 4 years than for 46% uptake at 3 years.

**Table 1.** Expected number of cancers screen detected, interval cancers, and cancers in non-participants per 10,000 persons invited for screening, over incident screens in the age range 50–70, by interscreening interval and percent uptake, in a homogeneous tumour population with MST of 4 years.

Interval in years (number of screens)	Detection mode	Uptake				
		46%	50%	60%	62%	70%
3 (6 screens)	Screen detected	222	240	288	300	336
	Interval cancers	149	163	196	200	228
	Cancers in non-participants	435	403	322	306	242
4 (5 screens	Screen detected	220	235	285	295	330
	Interval cancers	151	168	199	205	234
	Cancers in non-participants	435	403	322	306	242
5 (4 screens)	Screen detected	198	212	256	264	296
	Interval cancers	173	191	228	236	268
	Cancers in non-participants	435	403	322	306	242
6 (3 screens)	Screen detected	159	171	207	213	240
	Interval cancers	212	232	277	287	324
	Cancers in non-participants	435	403	322	306	242
7 (2 screens)	Screen detected	112	122	146	150	170
	Interval cancers	259	281	338	350	394
	Cancers in non-participants	435	403	322	306	242

MST: mean sojourn time.

Table 2 shows the number of screening invitations and examinations which would be carried out under the same combinations of interval and uptake. Compared with a 3-year interval and 46% uptake, a 4-year interval with 62% uptake would entail a cost in terms of 3400 more screening examinations taking place. However, there would be 10,000 fewer invitations in total over the entire screening lifetime. In addition, the number of screen-detected cancers per 1000 examinations would be higher with the longer round length, at around 9.5 cancers per 1000 compared to 8.0 per 1000 with three years.

**Table 2.** Numbers of incident screening invitations and screening examinations over the age range 51–70, by interval and percent uptake.

Interval (years)	Activity	Uptake				
		46%	50%	60%	62%	70%
3	Screening invitations	60,000	60,000	60,000	60,000	60,000
	Screening examinations	27,600	30,000	36,000	37,200	42,000
4	Screening invitations	50,000	50,000	50,000	50,000	50,000
	Screening examinations	23,000	25,000	30,000	31,000	35,000
5	Screening invitations	40,000	40,000	40,000	40,000	40,000
	Screening examinations	18,400	20,000	24,000	24,800	28,000
6	Screening invitations	30,000	30,000	30,000	30,000	30,000
	Screening examinations	13,800	15,000	18,000	18,600	21,000
7	Screening invitations	20,000	20,000	20,000	20,000	20,000
	Screening examinations	9200	10,000	12,000	12,400	14,000

Considered another way, if a certain fixed number of screening appointments were available with existing capacity, one could use the tabular data to suggest the optimum strategy of filling these appointments. For example, if we had approximately 30,000 incident screen appointments to fill, Tables 1 and 2 show that a 3-year interval with 50% uptake would require 60,000 invitations and result in 240 screen-detected cancers. A 4-year interval with 60% uptake would result in 285 cancers with 50,000 invitations.

### Mixture of fast growing and slow growing tumour populations

Tables 3 and 4 show the corresponding results for the slow-growing tumours with MST of 5 years, and the fast-growing with MST of 2.5 years, respectively. For the slow-growing tumour population, there is a substantial advantage for 62% uptake with a 4-year interval over 46% uptake with a 3-year interval, with 230 vs. 168 incident screen-detected cancers. Even for the fast-growing population with MST of 2.5 years there is an advantage, albeit a smaller one, for 62% uptake with a 4-year interval over 46% uptake with a 3-year interval, with 75 vs. 54 cancers incident screen detected.

**Table 3.** Expected number of cancers screen detected per 10,000 persons invited for screening, at incident screens over the age range 50–70, by interval and percent uptake, in a subpopulation with slowly progressing tumours (MST = 5 years).

Interval (years)	Screen-detected cancers	Uptake				
		46%	50%	60%	62%	70%
3	Screen detected	168	186	222	228	258
	Interval cancers	98	103	125	131	147
	Cancers in non-participants	313	290	232	220	174
4	Screen detected	170	185	220	230	260
	Total ages 51–70	96	104	127	129	145
	Cancers in non-participants	313	290	232	220	174
5	Screen detected	156	168	204	208	236
	Total ages 51–70	110	121	143	151	169
	Cancers in non-participants	313	290	232	220	174
6	Single screen	129	138	168	171	195
	Total ages 51–70	137	151	179	188	210
	Cancers in non-participants	313	290	232	220	174
7	Single screen	92	100	120	121	138
	Total ages 51–70	174	189	227	238	267
	Cancers in non-participants	313	290	232	220	174

MST: mean sojourn time.

**Table 4.** Expected number of cancers screen detected per 10,000 persons invited for screening, at a single incident screen, and over the age range 51–70, by interval and percent uptake, in a subpopulation with fast-growing tumours (MST = 2.5 years).

Interval (years)	Screen-detected cancers	Uptake				
		46%	50%	60%	62%	70%
3	Screen detected	54	60	72	78	84
	Interval cancers	66	70	85	84	99
	Cancers in non-participants	141	131	104	99	78
4	Screen detected	55	60	70	75	80
	Interval cancers	65	70	87	87	103
	Cancers in non-participants	141	131	104	99	78
5	Screen detected	48	52	60	64	72
	Interval cancers	72	78	97	98	111
	Cancers in non-participants	141	131	104	99	78
6	Screen detected	36	39	48	48	54
	Interval cancers	84	91	109	114	129
	Cancers in non-participants	141	131	104	99	78
7	Screen detected	24	28	32	34	38
	Interval cancers	96	102	125	128	145
	Cancers in non-participants	141	131	104	99	78

MST: mean sojourn time.

For the slow-growing tumour population, the benefit of 62% uptake over 46% was estimated to be outweighed by the increased round length of between 6 and 7 years. For the fast-growing tumours, the benefit was estimated as outweighed at between 5 and 6 years.

## Discussion

We estimated the number of cancers expected to be detected per 10,000 women invited at incident screens in the NHS breast screening programme, assuming underlying incidence, screening sensitivity and MST consistent with the literature and with results of the NHS Breast Screening Programme in England. We estimated the screen detection rates for a range of uptake rates and round lengths (inter-screening intervals). We took account of the fact that a greater round length would mean fewer incident screens between ages 51 and 70. We found that a greater total number of cancers would be screen detected, and therefore potentially detected at an earlier stage, for a round length of 4 years and uptake of 60% or more, than for a round length of 3 years and uptake of 50% or less. Specifically, a 3-year round length with an uptake rate of 46%, as observed in the recovery period in London, would imply 222 incident screen breast cancers per 10,000 invitations, detected over six incident screens up to age 70. On the other hand, a 4-year round length with 62% uptake, the latter observed in London prior to the pandemic, would imply 295 breast cancers incident screen detected (Table 1). As one would expect, combinations yielding larger numbers of screen-detected cancers gave correspondingly smaller numbers of symptomatic cancers, as a total of interval cancers and cancers in non-participants.

The increased cancer detection at 4-year intervals would require a larger number of screening examinations, but a smaller number of invitations (Table 2).

When we posited heterogeneity in the form of two tumour populations, one with a fast rate of progression from preclinical screen-detectable cancer to symptomatic disease and one with a slow rate, the same conclusion applied. Even for the population of fast-growing tumours, a 4-year round length with 62% uptake would yield more screen-detected cancers than a 3-year round length with 46% uptake, with 75 versus 54 incident screen-detected cancers, respectively (Table 4).

Clearly, improved uptake will imply improved breast cancer outcomes whatever the round length. There is evidence that timed appointments confer a substantial improvement in uptake over open invitations.^10^ As noted above, in London prior to the pandemic when timed appointments were in place, 62% uptake was observed, compared to 46% in the recovery period with open invitations as policy. It should be noted that the lower uptake of 46% is likely also to be due to reluctance to attend for fear of infection. What one might term psychosocial interventions, such as primary care endorsement and additional reminders, could improve uptake with either open invitations or timed appointments, but generally by smaller percentages.^6^

In the recovery period, the NHS Breast Screening Programme has opted for open appointments, meaning that those who wish to take up the offer of screening have to actively contact the service and book a screening appointment. This is in contrast to the practice prevailing before the pandemic of sending timed and located appointments with the invitation. The open appointments system tends to use the screening resource more efficiently in that fewer appointments are missed. However, it also tends to confer a lower uptake overall.^6,10^ While we do not advocate longer intervals between screens, the results here suggest that in the pandemic recovery period when the previous situation of 70% uptake and a maximum round length of 3 years is not available, some sacrifice of round length might be worthwhile. This might be particularly so if, for example, timed appointments might then be possible, conferring an increase in uptake and a corresponding increase in cancers screen detected.

As noted above, the results could be interpreted by taking the number of available appointments as a starting point. Where 30,000 slots are available for the population over the 21-year screening age range, screening 5000 women six times (3-year interval, 50% uptake) would yield 240 screen-detected cancers and require 60,000 invitations. Screening 6000 women five times (4-year interval, 60% uptake) would yield 285 screen-detected cancers and would require 50,000 invitations, and screening 7500 four times would yield 317 screen-detected cancers with 40,000 invitations. While in practice, we have no idea how to turn 50% uptake into 75%, a change to timed appointments could reasonably be expected to improve uptake from 50% to 60%.

The balance of screen-detected and interval cancers is affected by the total age range over which screening is offered, from age 50 to just before the 71^st^ birthday. For example, a 3-year interval in this age range implies six incident screens and seven 3-year intervals. On the other hand, a 4-year interval implies five incident screens, five 4-year intervals and one single-year interval. As a consequence, for very high or very low progression rates, there are actually slightly fewer interval cancers expected for 4-yearly screening within these age limits (Tables 3 and 4).

The main limitation of this work is that although the assumptions we made were consistent with peer-reviewed results and with previously published results from the NHS Breast Screening Programme, the values assumed for progression rates and screening test sensitivity may not be correct. However, we have run the calculations for a range of values, including considerably faster progression and considerably lower sensitivity. The qualitative result that a 4-year interval and 62% uptake yielded higher numbers of screen-detected cancers than a 3-year interval and 46% uptake remained the same in all cases (details available from the corresponding author).

Another limitation is that we have addressed only detection mode, not stage of disease. While there is a substantial survival difference between screen-detected and symptomatic tumours,^3^ an estimate of the effect on stage at diagnosis would arguably be a more sensitive measure of the likely effect on breast cancer outcomes.

Our estimated total numbers of incident cancers are based on an approximation but, bearing in mind that it excludes prevalent cancers at age 50, it is consistent with national incidence rates.^2^

In conclusion, there is a case for prioritising uptake, possibly at the expense of round length, in the recovery of the NHS Breast Screening Programme from the COVID-19 pandemic.

## Supplemental Material

sj-docx-1-msc-10.1177_09691413211066476 - Supplemental material for Recovery of the breast screening programme following pandemic-related delays: Should we focus on round length or uptake? Click here for additional data file. Supplemental material, sj-docx-1-msc-10.1177_09691413211066476 for Recovery of the breast screening programme following pandemic-related delays: Should we focus on round length or uptake? by Stephen W. Duffy, Sue Hudson, Daniel Vulkan, Thomas E. Duffy and Kathie Binysh in Journal of Medical Screening

## References

[1] Screening and Immunisations Team, NHS Digital. Breast screening programme: england 2018–19. Leeds: NHS Digital; 2020. https://digital.nhs.uk/data-and-information/publications/statistical/breast-screening-programme/england---2018–19 (last accessed 25th November 2021)

[2] https://www.cancerresearchuk.org/health-professional/cancer-statistics/statistics-by-cancer-type/breast-cancer/incidence-invasive#heading-Zero (last accessed 29th July, 2021)

[3] Massat NJ Sasieni PD Tataru D, et al. Explaining the better prognosis of screening-exposed breast cancers: influence of tumor characteristics and treatment. Cancer Epidemiol Biomarkers Prev 2016; 25: 479–487. 26646361 10.1158/1055-9965.EPI-15-0804

[4] Marmot MG, et al. The benefits and harms of breast cancer screening: an independent review. Br J Cancer 2013; 108: 2205–2240. 23744281 10.1038/bjc.2013.177 PMC3693450

[5] Richards M. Report of the Independent Review of Adult Screening Programmes in England. London: NHS England, 2019

[6] Duffy SW Myles JP Maroni R, et al. Rapid review of evaluation of interventions to improve participation in cancer screening services. J Med Screen 2016; 24: 127–145. 27754937 10.1177/0969141316664757 PMC5542134

[7] Day NE Walter SD. Simplified models of screening for chronic disease: estimation procedures from mass screening programmes. Biometrics 1984; 40: 1–14. 6733223

[8] Tabar L Vitak B Chen HH, et al. The Swedish Two-county trial twenty years later: updated mortality results and new insights from long term follow-up. Radiol Clin Nth Amer 2000; 38: 625–651. 10.1016/s0033-8389(05)70191-3 10943268

[9] Bennett RL Sellars SJ Moss RM. Interval cancers in the NHS breast cancer screening programme in england, wales and northern Ireland. Br J Cancer 2011; 104: 571–577. 21285989 10.1038/bjc.2011.3 PMC3049599

[10] Allgood P Maroni R Hudson S, et al. Effect of second timed appointments for non-attenders of breast cancer screening in england: a randomised controlled trial. Lancet Oncol 2017; 18: 972–980. 28522311 10.1016/S1470-2045(17)30340-6 PMC5489696

