# Supplementary material for: Recovery of the breast screening programme following pandemic-related delays: Should we focus on round length or uptake?
Source: J Med Screen. 2022 Jun;29(2):99–103. doi: 10.1177/09691413211066476 (PMC9087308; doi:10.1177/09691413211066476)
Supplement: sj-docx-1-msc-10.1177_09691413211066476 - Supplemental material for Recovery of the breast screening programme following pandemic-related delays: Should we focus on round length or uptake? [file sj-docx-1-msc-10.1177_09691413211066476.docx]

**Appendix** - number of cancers detected at an incident screen

We use the notation:

- sensitivity = S;
- the incidence of preclinical disease = λ_1_;
- progression rate from preclinical to symptomatic disease = λ_2_; and
- screening interval = t.

Assuming exponential distributions of time to incidence of preclinical disease and of progression from preclinical to symptomatic disease, the proportion of subjects detected with cancer at an incident screen is

$$R=S\left\{ \int_{0}^{t} \lambda_{1}e^{-\lambda_{1}s}e^{-\lambda_{2}\left( t-s \right)}ds+\left( 1-S \right)e^{-\lambda_{2}t}\int_{0}^{t} \lambda_{1}e^{-\lambda_{1}s}e^{-\lambda_{2}\left( t-s \right)}ds \right\}+$$

$${(1-S)}^{2}e^{-2\lambda_{2}t}\int_{0}^{t} \lambda_{1}e^{-\lambda_{1}s}e^{-\lambda_{2}(t-s)}ds+\ldots$$

The first term in the equation represents cancers newly arising since the last screen t years ago as preclinical cancers and not yet progressing to symptomatic disease. The second term represents cancers which were present at the previous screen but which were missed at that screen, and have remained preclinical since. The third screen represents those tumours which were missed at both the previous screen and the screen before that, and remain preclinical, and so on. Integrating, we obtain:

$$\int_{0}^{t} \lambda_{1}e^{-\lambda_{1}s}e^{-\lambda_{2}(t-s)}ds=\frac{\lambda_{1}(e^{-\lambda_{1}t}-e^{-\lambda_{2}t})}{(\lambda_{2}-\lambda_{1})}$$

This in turn gives:

$$R=\frac{{S\lambda}_{1}(e^{-\lambda_{1}t}-e^{-\lambda_{2}t})}{(\lambda_{2}-\lambda_{1})}\sum_{i} {(\left( 1-S \right)e^{-\lambda_{2}t})}^{i}$$

The limiting form of the summation is

$$\frac{1}{(1-(\left( 1-S \right)e^{-\lambda_{2}t})}$$

Thus

$$R==\frac{S\lambda_{1}(e^{-\lambda_{1}t}-e^{-\lambda_{2}t})}{(\lambda_{2}-\lambda_{1})(1-\left( 1-S \right)e^{-\lambda_{2}t})}$$
